# PI3K Inhibition Enhances Doxorubicin-Induced Apoptosis in Sarcoma Cells

**DOI:** 10.1371/journal.pone.0052898

**Published:** 2012-12-31

**Authors:** Diana Marklein, Ulrike Graab, Ivonne Naumann, Tiandong Yan, Rosalie Ridzewski, Frauke Nitzki, Albert Rosenberger, Kai Dittmann, Jürgen Wienands, Leszek Wojnowski, Simone Fulda, Heidi Hahn

**Affiliations:** 1 Institute of Human Genetics, University Medical Center, Goettingen, Germany; 2 Institute for Experimental Cancer Research in Pediatrics, University Frankfurt, Frankfurt, Germany; 3 Department of Pharmacology, University Medical Center, Mainz, Germany; 4 Department of Genetic Epidemiology, University Medical Center, Goettingen, Germany; 5 Department of Cellular and Molecular Immunology, University Medical Center, Goettingen, Germany; Faculté de médecine de Nantes, France

## Abstract

We searched for a drug capable of sensitization of sarcoma cells to doxorubicin (DOX). We report that the dual PI3K/mTOR inhibitor PI103 enhances the efficacy of DOX in several sarcoma cell lines and interacts with DOX in the induction of apoptosis. PI103 decreased the expression of *MDR1* and *MRP1*, which resulted in DOX accumulation. However, the enhancement of DOX-induced apoptosis was unrelated to DOX accumulation. Neither did it involve inhibition of mTOR. Instead, the combination treatment of DOX plus PI103 activated Bax, the mitochondrial apoptosis pathway, and caspase 3. Caspase 3 activation was also observed in xenografts of sarcoma cells in nude mice upon combination of DOX with the specific PI3K inhibitor GDC-0941. Although the increase in apoptosis did not further impact on tumor growth when compared to the efficient growth inhibition by GDC-0941 alone, these findings suggest that inhibition of PI3K may improve DOX-induced proapoptotic effects in sarcoma. Taken together with similar recent studies of neuroblastoma- and glioblastoma-derived cells, PI3K inhibition seems to be a more general option to sensitize tumor cells to anthracyclines.

## Introduction

Sarcomas are a heterogeneous group of malignant tumors of mesenchymal origin. More than 50 histological subtypes are known. Sarcomas comprised 1% of malignancies in adults but 15% in patients under 20 years old in North America in 2006 [1] and 8.5% of cancers in European patients aged 15–24 years diagnosed from 1990 to 1994 [2]. Thus, sarcomas are in general more prevalent in children. The most common histological sarcoma subtype in children is rhabdomyosarcoma (RMS) [3].

Sarcomas present a therapeutic challenge compared with other solid tumors both because of the limited success of traditional treatment approaches and because monitoring the response of sarcoma lesions to therapy is not straightforward [1]. The treatment of sarcomas usually comprises surgical resection, radiation treatment, and chemotherapy. Chemotherapy of sarcomas frequently involves anthracyclines, which are topoisomerase II (TOP2) inhibitors, in combination with other cytostatic drugs [4], [5]. One of the anthracyclines used in the therapy of sarcoma is DOX.

There remain unresolved issues associated with DOX use in sarcoma therapies. For example, the original phase II trials performed in RMS patients showed response rates between only 18% and 37% [6]–[8], while a response rate of 65% was reported in a more recent phase II study of patient with high-risk metastatic RMS [9]. Randomized phase III studies conducted by the North American Intergroup Rhabdomyosarcoma Study Group have investigated the addition of DOX to VAC (vincristine, actinomycin D and cyclophosphamide) chemotherapy for patients with Clinical Group III and IV RMS, but have failed to show any evidence of efficacy [10]–[12]. The lack of evidence of superiority of DOX with VAC over VAC alone and the potential for cardiotoxicity have limited the widespread use of DOX in the initial treatment of RMS. Since young children are particularly susceptible to anthracycline-induced cardiotoxicity, careful use of DOX is especially relevant [13], [14].

The efficacy of DOX in sarcoma therapies could be improved by combination with drugs other than those comprising VAC. To identify drugs enhancing antitumoral effects of DOX in sarcoma, we performed a screen using the sarcoma cell line HT1080. The screen included the proteasome inhibitor bortezomib, the DNA-demethylating agent 5-Aza-Deoxycytidin (5-Aza), the histone deacetylase inhibitor valproic acid (VPA) and the PPARy ligand pioglitazone. All these drugs have been shown to sensitize other tumor entities to antitumoral effects of TOP2A inhibitors, purportedly by increasing TOP2A expression levels [15]–[21]. Additionally, we used the dual PI3K and mTOR inhibitor PI103. This was due to the fact that sarcomas frequently show activation of PI3K/Akt/mTOR signaling [22], [23] and dual PI3K/mTOR inhibitors sensitize neuroblastoma and glioblastoma cells to DOX-induced apoptosis [24], [25].

Our present study was prompted by the observation that PI103 interacts with DOX in the induction of apoptosis and in activation of caspase 3 in 3 different sarcoma cell lines. Since these proapoptotic effects were in contrast to recent data for the dual PI3K/mTOR inhibitor NVP-BEZ23 in sarcoma cells, we investigated the underlying mechanism in more detail.

## Materials and Methods

### Reagents

DOX and bortezomib were dissolved in 0.9% NaCl. 5-Aza and VPA were dissolved in PBS and pioglitazone, PI103, zVAD.fmk and LY294002 in DMSO, and everolimus in ethanol. GDC-0941, a PI103 analog [26], was obtained from Genentech Inc. (San Francisco, California, USA) and dissolved in Methylcellulose-Tween-Solution (MTS) or DMSO for *in vivo* or *in vitro* application, respectively.

### Cell Culture

The human RMS cell line RD and the human sarcoma cell line HT1080 were obtained from ATCC. The murine RMS cell line TP5014 was a gift from Professor Torsten Pietsch (Department of Neuropathology, University of Bonn, Germany). TP5014 is a stable murine RMS cell line derived from a RMS of a *Ptch^neo12/+^* mouse [27] with the consideration of all necessary legal requirements (no ethics committee approval was required; personal communication from Torsten Pietsch). All cell lines were cultured in DMEM, 10% FCS, and 1% penicillin/streptomycin. Medium used to culture HT1080 cells was additionally supplemented with 20 mM Hepes, 10 mM sodium pyruvate and 4% (v/v) non-essential amino acids.

For gene expression analysis and determination of apoptosis 100 000 cells/well were seeded in 6-well-plates. For Caspase-Glo® 3/7 and BrdU incorporation assay 5,000 cells/well were seeded in 96-well-plates. Cells were allowed to settle for 24 h. After washing, cells were incubated for 24 h with medium supplemented with drugs or solvent as indicated in the respective experiments. For pretreatment of the cells with PI103 cells were pretreated with the drug for 12 h and DOX was added to the same medium for additional 24 h.

Cell proliferation was measured after BrdU-pulsing for the last 24 h using a Cell Proliferation BrdU ELISA (Roche Diagnostics GmbH, Mannheim, Germany). Drug-induced BrdU-incorporation is presented as the percentage of the incorporation measured in time-matched vehicle-treated controls taken as 100%.

Apoptosis was determined of cells stained with Annexin V-FITC (BD Biosciences, Heidelberg, Germany) and To-Pro-3 iodide (PI, Invitrogen GmbH, Karlsruhe, Germany) on a FACScan system (BD Biosciences).

Activity of caspase 3 and 7 was measured using the Caspase-Glo® 3/7 Assay (Promega). The luminescence intensity is shown as the fold-induction over the control value, which was set to 1.

Data shown are representative for 2 to 8 independent experiments performed as duplicates.

### Quantification of Intracellular Doxorubicin

To measure the intracellular amount of DOX quantitatively, RD or HT1080 cells were seeded in 6-well-plates (10^5^ cells/well). 24 h later, DOX alone, DOX combined with PI103, or PI103 alone were added. After incubation for 24 h, the drug-containing culture medium was discarded and the cells were washed with PBS. Cells were gently harvested using accutase and cell pellets were obtained by low-speed centrifugation (300 g, 5 min, 4°C). After washing with PBS, cells were collected and DOX fluorescence was measured by flow cytometry using a FACSCalibur (BD Biosciences, Heidelberg, Germany). Excitation wavelength was 488 nm, and emission wavelength was 530 nm. Intracellular DOX was quantified in at least 10 000 cells from each sample.

### RNA Extraction, Reverse Transcription and Quantitative RT-PCR-analyses

Total RNA was isolated using TRIzol Reagent (Invitrogen GmbH, Karlsruhe, Germany) according to the manufacturer’s instruction. cDNA was synthesized using Superscript II and random hexamers (Invitrogen, Karlsruhe, Germany). Quantitative RT-PCR of target cDNAs was performed using SYBR-green based assays. Primer pairs used for amplification of *MDR1* (5′-GTGGTGGGAACTTTGGCTG/5′-TACCTGGTCATGTCTTCCTCC) and *MRP1* (5′-ATGTCACGTGGAATACCAGC/5′-GAAGACTGAACTCCCTTCCT) were intron-spanning. Amplification of *18S rRNA* (5′- CGCAAATTACCCACTCCCG/5′-TTCCAATTACAGGGCCTCGAA) served to normalize any inter-sample differences in the efficiency of reverse transcription. Real time quantitative RT-PCR analysis was carried out using the ABI Prism HT 7900 Detection System instrument and software (Applied Biosystems, Darmstadt, Germany). The data shown are representative for at least five independent experiments. Each sample of every experiment was measured as a triplicate.

### Western Blot Analysis

Generally, cells were lysed in a buffer containing 30 mM Tris-HCl pH 7.4, 150 mM NaCl, 1% Triton X-100, 10% Glycerol, 500 µM PMSF, 2 mM DTT, and a protease inhibitor cocktail. For detection of MRP1, cells were lysed in a RIPA buffer containing 50 mM Tris-HCl (pH 7.4), 150 mM NaCl, 1 mM EDTA, 1% NP-40, 0.25% Na-Deoxycholat and the protease inhibitor cocktail. Protein concentrations were determined by the Pierce Protein BCA Assay Kit (Themo Fisher Scientific, Rockford, USA). Antibodies used to detect the individual target proteins are shown in Table S1.

For detection of active Bax, cells were lysed in CHAPS lysis buffer (10 mM HEPES (pH 7.4); 150 mM NaCl; 1% CHAPS). A total of 500 µg protein was immunoprecipitated with 2 µg mouse anti-Bax antibody (6A7, Sigma) and 5 µl Dynabeads Pan Mouse IgG (Dako, Hamburg, Germany). The precipitate was analyzed by western blotting using the BaxNT antibody (Upstate Biotechnology) (see also [28]).

All Western blots shown are representative of at least two independent experiments.

### Determination of Cytochrome c Release

Cytochrome c release was determined as previously described [29], [30].

### *In vivo* RD Xenograft Model and Treatment with GDC-0941 and/or DOX

Nude mice used in the study were handled in accordance with the German animal protection law and the experiments were approved by the Niedersächsisches Landesamt für Verbraucherschutz und Lebensmittelsicherheit (permit number: 33.42502-04-09508). Aliquots of 2×10^6^ viable RD cells in 200 µl PBS/Matrigel (1∶1) were injected subcutaneously (s.c.) into the flank region of the mice. Tumors were measured twice weekly with calipers, and tumor volumes were calculated by the formula [length×width×(height/2)] [31]. The body weight and general physical status of the animals were recorded every 3 days. Treatment of the animals bearing sarcoma xenografts started when the tumor’s volume reached 30–60 mm^3^.

GDC-0941 was administered orally at the recommended daily dose of 75 mg/kg body weight for a total of 22 days. DOX at 1.2 mg/kg body weight was administered intraperitoneally (i.p.) every third day on days 1, 4, 7, 10, 13, 16, 19 and 22. This continuous low-dose schedule has been shown to moderately but significantly inhibit the growth of RD xenografts in other studies [32]. Combined treatment comprised these drugs at the same doses as in individual treatments. Control animals bearing s.c. flank tumors were treated with MTS and 0.9% NaCl following the same schedules.

At the end of the study, tumor nodules were carefully dissected and fixed in a 4% PFA solution for further analysis. In addition, tumor regrowth was monitored over a period of 20 days after withdrawal of the drugs.

### Immunohistochemical Analysis

For analysis of caspase 3 activity in the xenotransplants, paraffin sections were boiled in citrate buffer (pH 6.0) for 45 min. The endogenous peroxidase was quenched with 3% hydrogen peroxide. Staining was done with anti-active caspase 3 (R&D Systems; 1∶500 in 10% Casein in TBS, pH 7.4) followed by incubation with rabbit/mouse Envision HRP ready (DakoCytomation, Hamburg, Germany). Signals were detected with AEC solution. Thousand cells of each tumor were evaluated for positive signals. The results of 7–9 tumors for each treatment group were summarized to calculate the percentage of positive cells. For TUNEL (Terminal deoxynucleotidyl transferase dUTP nick end labeling) assay, the DeadEnd Colorimetric TUNEL System (G7130, Promega) was used.

### Statistical Analysis

Unless indicated otherwise, statistical differences for all experiments using more than two treatment schemes were analyzed using Tukey’s test in conjunction with ANOVA. When comparing 2 samples, statistical differences were analyzed using Student’s *t*-test. Data was considered significant when *P*<0.05. Interaction between PI103 and DOX was analyzed by the combination index (CI) method using CalcuSyn software (Biosoft). CI <0.9 indicates synergism, 0.9–1.1 indicates additivity, and >1.1 indicates antagonism.

## Results

### Effects of DOX in Combination with 5-Aza, VPA, Bortezomib, Pioglitazone, or PI103 in Sarcoma Cells

The effects of DOX alone or in combination with candidate drugs on proliferation, apoptosis, and caspase 3/7 activity (as measured by Caspase-Glo® 3/7 Assay) were investigated in the sarcoma cell line HT1080. DOX effects were studied at a concentration of 1 µM, which is within the plasma concentration of this drug in humans [33], [34].

As shown in Table 1, treatment of the cells with 1 µM DOX resulted in a significant proliferation inhibition by 80%. In addition, 1 µM DOX significantly increased the numbers of Annexin V-positive cells to 27% (solvent-treated cells 3%) and caspase 3/7 activity to 2-fold over the control.

**Table 1:** PI103 sensitizes HT1080 cells to DOX-induced apoptosis.

Treatment		BrdU-incorporation (% over solvent)	Annexin V positive cells (%)	Increase caspase 3/7 activity (over solvent)
Drug	Concentration			
**DOX**	1 µM	−80%*	27%*	2-fold*
**5-Aza**	5 µM	+80%*	3%	1.4-fold
**DOX/5-Aza**	1 µM/5 µM	−60%^#^	31%*	2.1-fold
**VPA**	2 mM	+50%*	3.6%	1.8-fold
**DOX/VPA**	1 µM/2 mM	−70%*	33.7%*	2.8-fold*
**bortezomib**	10 nM	−70%*	10%	4.9-fold*
**DOX/Borte**	1 µM/10 nM	−**90%^#^**	40%*	1.5-fold
**pioglitazone**	10 µM	+10%	2.4%	1-fold
**DOX/Pio**	1 µM/10 µM	−90%*	**42.5%^#^**	1.4-fold
**PI103**	1 µM	−10%*	2.5%	0.9-fold
**DOX/PI103**	1 µM/1 µM	−90%*	**53%^#^**	**5-fold^#^**

Proliferation of HT1080 cells was measured by BrdU incorporation. Shown is the increase or decrease in comparison to vehicle-treated cells. Apoptosis was analyzed by FACS of Annexin V positive cells and caspase 3/7 activity was measured by Caspase-Glo® 3/7 assay. Statistical significance was assessed by ANOVA/Tukey’s method. *P<0.05 compared to cells treated with solvent; #*P*<0.05 compared to cells treated with either drug alone. Numbers in bold indicate cases, where given drug combination *inhibited proliferation* or *induced apoptosis* to a larger extent than either drug alone.

Candidate drugs were applied to HT1080 cells at concentrations described for other cell culture experiments [15]–[19]. Whereas treatment of the cells with 5-Aza (5 µM) and VPA (2 mM) significantly induced the proliferation rate of the cells, bortezomib (10 nM) and PI103 (1 µM) significantly reduced it. None of the drugs had a significant effect on the number of Annexin V-positive cells (Table 1). However, bortezomib significantly induced caspase 3/7 activity.

Next, the drugs were combined with DOX. As shown in Table 1, DOX-induced proliferation inhibition was significantly enhanced by bortezomib. The enhancement by PI103 was of borderline significance (*P*<0.08). DOX-related apoptosis was also enhanced by both pioglitazone and PI103, as indicated by the significant increase of Annexin V-positive cells. In addition, PI103 was able to enhance DOX-mediated induction of caspase 3/7 activity.

In summary, the dual PI3K/mTOR inhibitor PI103 significantly affected 2 of the measured parameters of apoptotic cell death when combined with DOX, i.e. it significantly enhanced the proapoptotic effects of DOX measured by Annexin V staining and by Caspase-Glo 3/7 assay. Furthermore, it enhanced DOX-induced antiproliferative effects (*P*<0.08). Therefore, we focused on this compound as a promising combination partner for DOX and investigated the generality of the combined effects towards sarcoma cells by including the TP5014 and RD cell lines derived from murine and human RMS, respectively.

### PI103 Interacts with DOX in the Induction of Apoptosis in Sarcoma Cells

**Figure 1:**
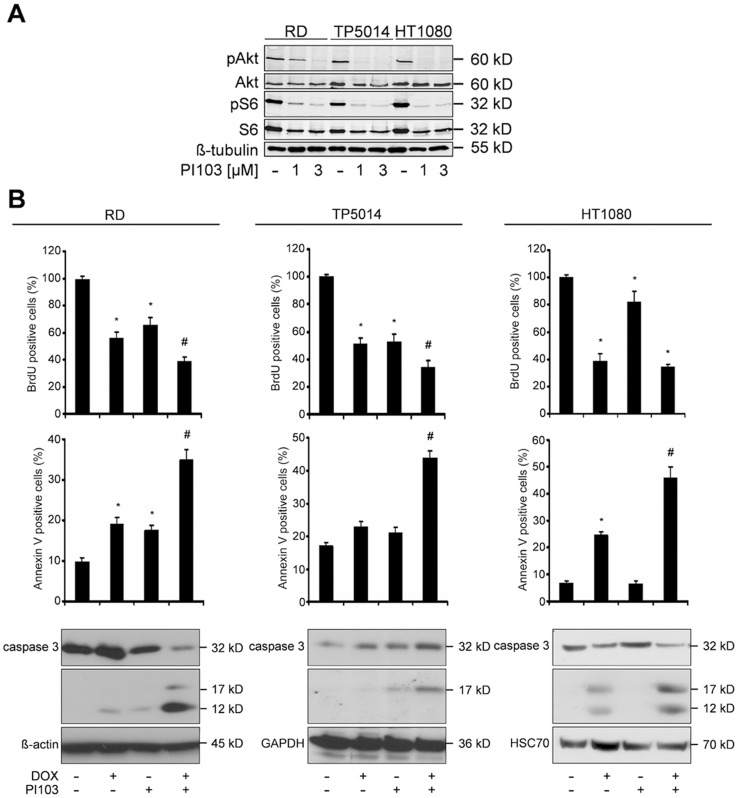
PI103 sensitizes RD cells to DOX-induced apoptosis. **A)** Levels of phospho-Akt, Akt, phospho-S6 ribosomal protein and S6 ribosomal protein were measured by Western blot analyses in RD, TP5014 and HT1080 cells after treatment with 1 µM or 3 µM PI103 as indicated. **B)** Upper panel: Proliferation was estimated by BrdU incorporation after treatment with 0.5 µM DOX and/or 3 µM PI103. Middle panel: Apoptosis was analyzed by FACS after treatment with 1 µM DOX and/or 3 µM PI103 or solvent. Data represent mean+SEM of at least three independent experiments performed in triplicates. Comparisons were made with ANOVA/Tukey’s testing. *P<0.05 compared to cells treated with solvent; #*P*<0.05 compared to cells treated with either drug alone. Lower panel: Protein levels of phospho-Akt, Akt and caspase 3 in cells treated with 1 µM DOX and/or 3 µM PI103 or respective solvents.

We first assessed whether the dual PI3K/mTOR inhibitor PI103 blocks PI3K/Akt/mTOR signaling in the cell lines examined by assessing phosphorylation of Akt and S6 ribosomal protein taken as surrogate readouts for the activity of PI3K and mTOR, respectively. As shown in Figure 1A, both RMS cell lines exhibit intact PI3K/mTOR signaling, as do HT1080 cells. This is evidenced by phosphorylation of Akt and S6, which was efficiently decreased by PI103 (Figure 1A). Treatment with 3 µM PI103 almost completely inhibited Akt phosphorylation in all 3 cell lines. Therefore, this dose was applied in all consecutive experiments.

Besides dephosphorylation of S6 we also observed a decrease in the total amount of S6 after incubation with PI103. This was in agreement with previously published data shown for the dual PI3K/mTOR inhibitor NVP-BEZ235 in breast cancer cells [35].

Next we assessed the antiproliferative and proapoptotic effects of 3 µM PI103 alone or in combination with DOX in sarcoma cells. In order to avoid overlooking potential cooperative antiproliferative effects, the DOX concentration was lowered to 0.5 µM in BrdU assays. The proliferation rate of all cell lines was decreased by approximately 50% in comparison to untreated controls (see Figure 1B). Apoptosis was measured at 1 µM DOX.

Both DOX and PI103 alone consistently reduced cell proliferation in all 3 sarcoma cell lines (Figure 1B). When both drugs were combined, a significant enhancement of DOX-induced antiproliferative effects was seen in rhabdomyosarcoma cells (Figure 1B). A calculation of the CI in RD and HT1080 cells revealed that PI103 synergistically cooperated with DOX to inhibit proliferation (CI = 0.769 and CI = 0.766, respectively). In respect of apoptosis, DOX treatment resulted in a significant increase of Annexin V positive RD and HT1080 cells. After PI103 treatment, a significant increase was only seen for RD cells (Figure 1B). This was different when both drugs were combined. As shown in Figure 1B, the combination treatment resulted in a strong and significant increase of Annexin V positive cells in all cell lines examined. When we distinguished between early (Annexin V^+^ PI^−^) and late (Annexin V^+^ PI^+^) apoptotic cells, we found that the combination treatment increased either of these cellular subsets to approximately the same extent (Figure S1). Furthermore, incubation with the broad-range caspase inhibitor zVAD.fmk almost completely blocked apoptosis upon combined treatment with PI103 and DOX, showing that apoptosis was caspase-dependent (Figure S2). Indeed, in all 3 cell lines, the increase in Annexin V positive cells was accompanied by an enhancement of caspase 3 activation, which was revealed by the occurrence of cleaved and active caspase 3 fragments (Figure 1B).

Together these data demonstrated that the combined treatment with PI103 and DOX results in enhancement of apoptosis of sarcoma cells. In the following, we elucidated the mechanism of the interaction of DOX and PI103 in apoptosis in more detail using RD cells.

### PI103 Inhibits the Expression of DOX Efflux Transporters and Increases Intracellular DOX Concentrations

The PI3K/Akt signaling has been reported to activate the expression of the DOX efflux transporters MDR1 (P-gp) in breast and gastric cancer cells [36], [37] and of MRP1 in AML [38] and in prostate carcinoma cells [39]. Based on these observations we hypothesized that inhibition of PI3K/Akt activity decreases the expression of MDR1 and MRP1, leading to DOX accumulation in the tumor cells.

**Figure 2:**
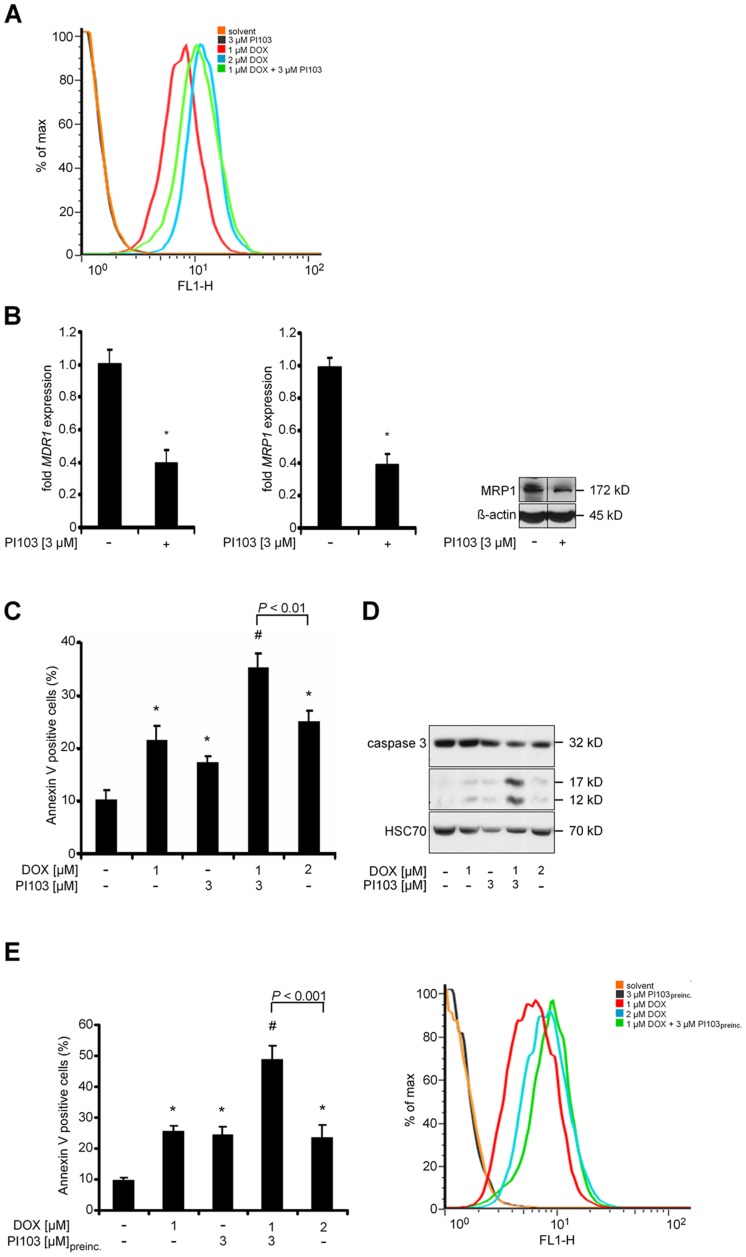
PI103-induced DOX accumulation does not account for cooperative proapoptotic effects in RD cells. **A)** Intracellular DOX autofluorescence after treatment with 1 µM DOX (red line), 3 µM PI103 (black line), 2 µM DOX (blue line), 1 µM DOX plus 3 µM PI103 (green line) or solvent (orange line) at 530 nm. The data are expressed as the percentage of the maximum (max) number of positive cells. **B)** Expression levels of *MDR1* (left panel) and *MRP1* (middle panel) after treatment with 3 µM PI103 in relation to solvent treated cells (set = 1). Data represent mean+SEM of at least five independent experiments performed in triplicates. *P<0.05 by Students *t*-test. Protein level of MRP1 after treatment with 3 µM PI103 or the solvent (right panel). **C)** Annexin V positive RD cells were analysed after treatment with 1 µM DOX, 3 µM PI103, 1 µM DOX plus 3 µM PI103, 2 µM DOX or the solvent. Data represent mean+SEM. Comparisons were made with Students *t*-test. **D)** Cells were treated as in C and caspase 3 was assessed by Western blot analyses. **E)** Annexin V positive RD cells (left panel) and intracellular DOX autofluorescence (right panel) after a 12-hours pretreatment (preinc.) with 3 µM PI103 and subsequent addition of 1 µM DOX for additional 24 h. Annexin V data represent mean+SEM. Comparisons were made with Students *t*-test.

Indeed, as revealed by FACS analysis in the living cell fraction, the treatment with 1 µM DOX in conjunction with 3 µM PI103 resulted in intracellular DOX accumulation (Figure 2A). The accumulation approximately equated that caused by treatment with 2 µM DOX. Similarly, in HT1080 cells, the DOX-specific fluorescence after combination treatment with 1 µM DOX plus 3 µM PI103 equated that caused by incubation with 3 µM DOX (data not shown).

The accumulation of DOX correlated with changes in the expression of MDR1 and MRP1. As shown in Figure 2B, PI103 resulted in a 60% decrease in the transcription of both *MDR1* and *MRP1* mRNA (Figure 2B; left and middle panel). The PI103-mediated decrease in *MRP1* expression was confirmed on the protein level (Figure 2B; right panel). Unfortunately, the analysis of MDR1 protein was impossible due to very low basal protein expression levels in RD cells.

### PI103-mediated Intracellular DOX Accumulation is not Responsible for the Combined Proapoptotic Effect of PI103 and DOX

We next investigated if the PI103-associated DOX accumulation was responsible for the combined effects of the drugs on apoptosis. To this end we incubated RD cells with 2 µM DOX. This resulted in an intracellular DOX accumulation similar to that following an incubation with 1 µM DOX plus 3 µM PI103 (see above). Although the treatment with 2 µM DOX increased the percentage of Annexin V positive cells when compared to 1 µM DOX, the increase was significantly smaller than that observed after treatment with 1 µM DOX plus 3 µM PI103 (Figure 2C). In addition, 2 µM DOX did not enhance caspase 3 activity as did the combination treatment (Figure 2D). Similar results were obtained for HT1080 cells (data not shown).

We also analyzed if an additional 12-hours pretreatment with PI103 enhanced sensitization of sarcoma cells to DOX-induced anticancer effects. Like the 24-hours incubation, a 36-hours incubation with PI103 alone significantly induced apoptosis of RD cells when compared to untreated cells (Figure 2E, left panel). The pre-exposure further strengthened the sensitization of RD cells to DOX-mediated anticancer effects. This was not only evident by a stronger antiproliferative effect (Figure S3A; for HT1080 and TP5014 cells see Figure S3B and S3C), but also by a further increase of Annexin V-positive cells (approximately 5-fold compared to the control; see Figure 2E, left panel) when compared to the increase of Annexin V positive cells after the 24-hours co-incubation (approximately 3.5-fold compared to the control; see Figure 2C, left panel). However, the preincubation with PI103 did not result in a further increase in DOX accumulation (Figure 2E, right panel). Together, these data indicated that the enhancement of DOX-induced apoptosis by PI103 cannot solely be explained by PI103-associated DOX accumulation.

### Combination Treatment with PI103 and DOX Induces Bax Activation and Enhances Cytochrome c Release

PI103 has been recently reported to cooperate with DOX to shift the ratio of pro- and antiapoptotic Bcl-2 proteins finally resulting in activation of the proapoptotic protein Bax [25]. Activated Bax translocates to the mitochondrial membranes, where it causes the loss of mitochondrial membrane potential and subsequent cytochrome c release and caspase activation [40]. Activation of Bax goes along with a conformational change in its N terminus in the cytosol, which precedes Bax translocation to the mitochondria and which can be detected with the active conformation-specific anti-Bax antibody 6A7 [28], [41].

We first investigated the effect of PI103 and DOX on Bax activation by immunoprecipitation of protein lysates with anti-Bax antibody 6A7 and subsequent analysis by western blotting using BaxNT antibody as previously described [28]. Secondly, we investigated the related cytochrome c release by means of FACS analysis using the cytochrome c-specific antibody 7H8.2C12. This antibody does not detect cytosolic cytochrome c after apoptosis induction [30]. Therefore, the reduction in the cytochrome c signal reflects mitochondrial cytochrome c release and early onset of apoptosis.

**Figure 3:**
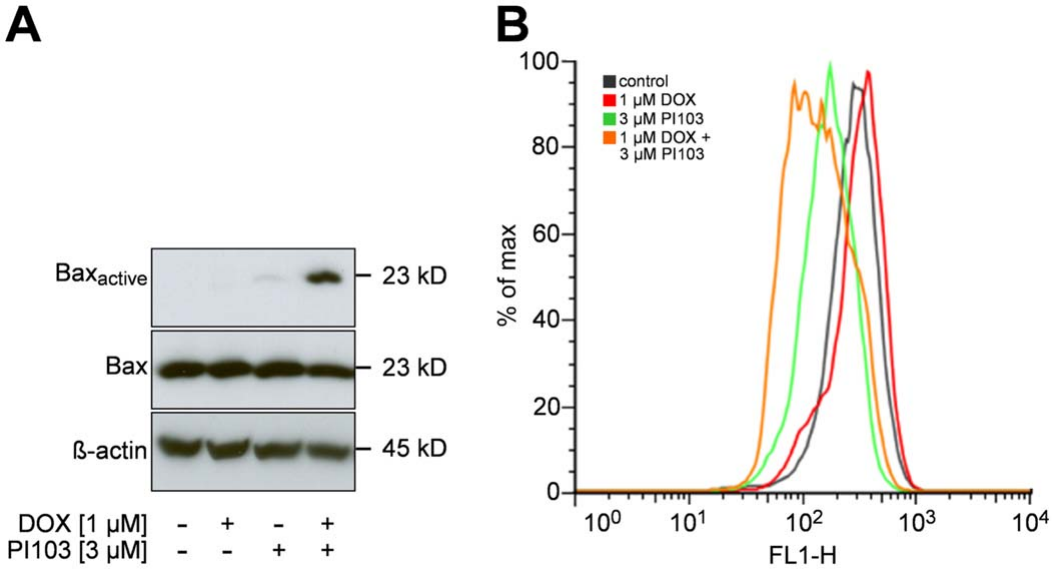
PI103 and DOX cooperate in Bax activation and cytochrome c release. RD cells were treated with 1 µM DOX and/or 3 µM PI103 or the solvent. **A)** Activation of Bax was analysed by immunoprecipitation. **B)** Cytochrome c release was assessed by FACS at 530 nm (red line: 1 µM DOX; green line: 3 µM PI103; orange line: 1 µM DOX plus 3 µM PI103; black line: solvent). The data are expressed as the percentage of the maximum (max) number of positive cells and represent one of three independent experiments, measured in duplicates.

As revealed by Western Blot analysis, DOX did not alter Bax activity, whereas PI103 marginally changed the conformation of Bax. However, the effect on Bax activation was strongly enhanced when both drugs were combined (Figure 3A). Bax activation after PI103/DOX co-treatment was also demonstrated in TP5014 and HT1080 cells (Figure S4A and S4B). A similar result was seen when cytochrome c release into the cytosol was studied. Whereas monotherapy with PI103 had a moderate effect on cytochrome c release, the combination with DOX strongly enhanced this effect (Figure 3B).

Together these data demonstrate that the combined effect of PI103 plus DOX involves Bax activation and cytochrome c release.

### The Specific PI3K Inhibitor LY294002, but not the mTOR Inhibitor Everolimus, Enhances DOX-induced Apoptosis

**Figure 4:**
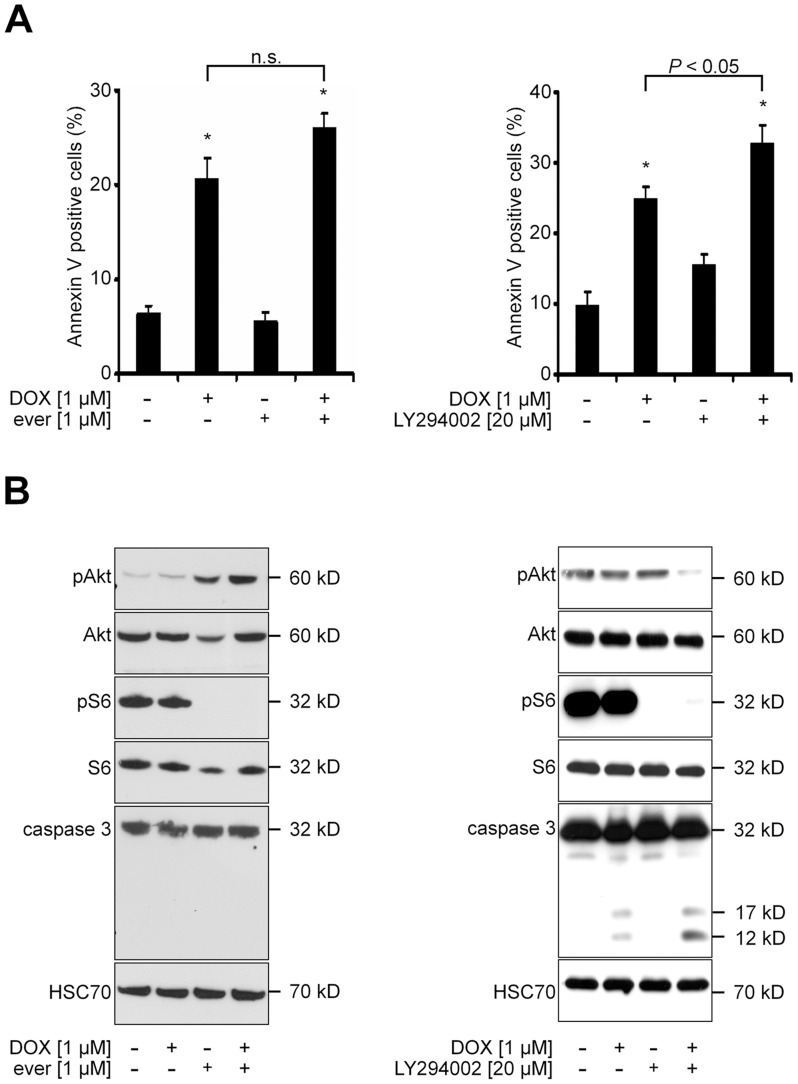
Inhibition of mTOR does not sensitize RD cells to DOX-induced apoptosis. **A) and B)** RD cells were treated for 24h with 1 µM DOX and/or 1 µM everolimus (ever; respective left panels) or with 1 µM DOX and/or 20 µM LY294002 (respective right panels) or solvent. **A)** Annexin V positive RD cells were analysed by FACS. Data represent mean+SEM of two independent experiments performed in duplicates. n.s. not significant by Students *t*-test. **B)** Western blot analyses.

Next, we investigated to what extent the combined proapoptotic effects of PI103 plus DOX were mediated by mTOR or by PI3K. To this end, we treated RD cells with the specific mTOR inhibitor everolimus or with the specific PI3K inhibitor LY294002 alone or in combination with DOX. The data shows that everolimus neither significantly increased the DOX-induced percentage of Annexin V positive cells nor induced caspase activity (Figure 4A left panel, and Figure 4B left panel, respectively). This was not due to lack of activity of everolimus, because the drug efficiently reduced phosphorylation of the mTOR target S6 (Figure 4B left panel). However, we also observed that everolimus increased phosphorylation of Akt. This phenomenon has been demonstrated previously by several groups in several cancer cell lines including RD cells [35], [42], [43]. In contrast, LY294002 significantly increased DOX-induced apoptosis as revealed by Annexin V labeling (Figure 4A right panel) and by increased caspase activity (Figure 4B right panel). The combined proapoptotic effect also correlated with Akt dephosphorylation, which was not seen upon single drug treatment.

Together the data show that mTOR inhibition does not significantly affect DOX-induced apoptosis and suggest that the proapoptotic effects of a combination treatment using DOX plus a dual PI3K/mTOR inhibitor such as PI103 mainly involves inhibition of PI3K, but not of mTOR.

### GDC-0941 Inhibits RD Growth *in vivo*, but does not Enhance DOX-induced Antitumor Effects Despite Increased Caspase 3 Activity

The solubility of PI103 is limited and the compound is metabolized extensively. Therefore, its use in *in vivo* studies is limited [26]. An optimization program focused on improving pharmaceutical properties of PI103 has led to the development of GDC-0941. In comparison to PI103, GDC-0941 is much more PI3K-specific, whereas its effect on mTOR is negligible [26], [44]. In addition, GDC-0941 is currently used in clinical trials in cancer patients (e.g. NCT00876109; NCT00960960). Since the involvement of mTOR in the enhancement of DOX-induced apoptosis in RD cells was unlikely (see Figure 4A and 4B), we precluded PI103 from *in vivo* studies and instead treated RD-transplanted nude mice with GDC-0941 and/or DOX (for cooperative effect of GDC-0941 plus DOX in the induction of apoptosis in RD cells see Figure S5).

**Figure 5:**
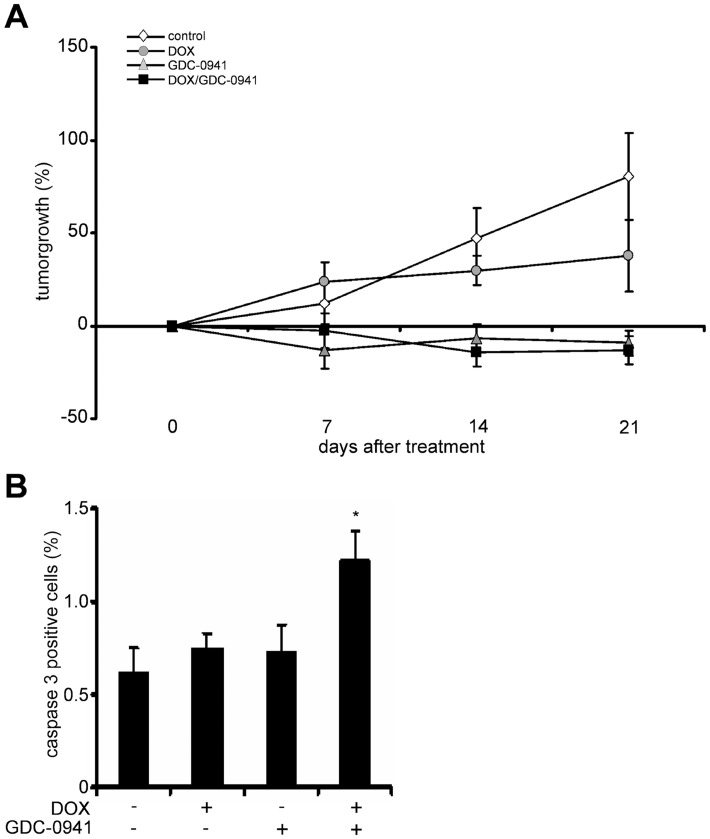
GDC-0941 inhibits tumor growth and increases DOX-mediated effects on caspase 3 activity. **A)** Inhibition of RD tumor growth (in %) in nude mice treated with 1.2 mg/kg DOX (i.p. every third day for 22 days; n = 19), 75 mg/kg GDC-0941 (orally every day for 22 days; n = 9), the combination of both drugs (n = 12) at the time points indicated. Vehicle-treated animals served a controls (n = 18). **B)** Caspase 3 positive cells (in %) in tumors of nude mice isolated after 22 days of treatment with DOX and/or GDC-0941 or solvent. Data represent mean+SEM. *P<0.05 by ANOVA/Tukey’s testing.

In the experiment presented in Figure 5A, the animals were treated with the drugs for a period of 22 days. In order to avoid overlooking transient effects on apoptosis, the tumors of the animals were harvested within 24 h after the last application of either drug. As shown in Figure 5A, monotherapy with either DOX or GDC-0941 over a period of 22 days significantly attenuated tumor growth (Figure 5A; Table S2), without causing any obvious side effects. Compared to the continuous low-dose application of DOX, GDC-0941 had a more pronounced effect on tumor growth inhibition. In fact, GDC-0941 completely stopped tumor growth after 7 days of treatment (Figure 5A; Table S2). However, the antitumor effects were not increased when tumors were additionally treated with DOX (Figure 5A, Table S2). A similar result was observed when a lower dose of GDC-0941 (i.e. 1/3 of the initial dose) was combined with DOX (Table S2). Moreover, tumor regrowth was not different between the treatment groups after withdrawal of the drugs (Table S3). Although immunohistochemical analysis of paraffin-embedded tumor sections did not reveal any significant difference in TUNEL positive cells (data not shown), the combination increased the percentage of cells positive for active caspase 3 when compared to application of either DOX or GDC-0941 alone (Figure 5B). These data demonstrated that the combination therapy consisting of DOX plus GDC-0941 elevates caspase 3 activity *in vivo.* However, this increase in caspase 3 activity caused by the combination treatment does not translate into a cooperative suppression of tumor growth.

## Discussion

DOX is a potent anticancer drug employed in the therapy of several solid tumors including sarcoma. Its mode of action is not completely understood, but involves inhibition of TOP2A and intercalation into DNA, which finally results in double strand breaks and inhibition of DNA replication [45], [46]. DOX also interferes with apoptotic processes and mediates the release of cell-damaging radicals [47].

Despite being one of the drugs of choice in sarcoma treatment, DOX sometimes does not show any efficacy [10]–[12]. In addition, it has a very high potential for cardiotoxicity, which is a limiting factor of DOX therapy, particularly in children [13], [14]. Therefore, this drug remains a controversial treatment option in sarcoma, especially in RMS, the most common sarcoma of childhood.

We aimed at enhancing the efficacy of DOX in sarcoma cells. We first applied several drugs with reported DOX-sensitizing capacity in other cancer cells lines. 5-Aza, VPA, pioglitazone and bortezomib were promising candidates as these drugs can enhance TOP2A expression, which is an important determinant in DOX responsiveness [48]. Indeed, in a pre-screen using HT1080 cells, bortezomib and pioglitazone enhanced DOX-mediated antiproliferative and proapoptotic effects, respectively. Although these drugs certainly deserve more investigations with respect to the enhancement of DOX-induced antitumoral responses (e.g. pretreatment of the cells with the drugs; application of the drugs at a higher dose; combination with lower or higher DOX concentrations), we focused on PI103, which simultaneously enhanced DOX-induced proliferation inhibition, induction of apoptosis, and activation of caspase 3 in all three sarcoma- and RMS-derived cell lines investigated.

The PI3K/Akt/mTOR signaling pathway represents a promising target for therapeutic intervention, since it is abnormally activated in many different human sarcoma types [1], [49]. The current focus in the clinics is on inhibition of mTOR. Besides monotherapy with mTOR inhibitors, several combinations with other agents including anthracyclines are being investigated in sarcomas. Among these are ridaforolimus and DOX (NCT00288431), or temsirolimus plus pegylated liposomal DOX in resistant solid malignancies or recurrent sarcoma (NCT00703170; NCT00949325).

However, pure mTOR inhibition (alone or in combination with other cytostatics) should be carefully reconsidered, because inhibition of mTOR results in activation of Akt in some tumor entities including RMS [42], [43], [50] (this study, see Figure 4). Therefore, the better choice for the treatment of these tumors could be either dual PI3K/mTOR inhibitors targeting both PI3K (thus preventing Akt activation) and mTOR, or pure PI3K inhibitors.

Dual PI3K/mTOR inhibitors can sensitize a variety of cancer cell lines to the treatment with DOX, but only one study reports combined treatment effects in sarcoma. In the latter study, the combination of the dual PI3K/mTOR inhibitor NVP-BEZ235 plus DOX resulted in a potentiation of antiproliferative effects *in vitro* [51]. However, neither a NVP-BEZ235-mediated enhancement of DOX-induced proapoptotic effects nor antitumoral effects in *in vivo* experiments were reported [51].

Our results now show that the PI103-mediated sensitization of sarcoma cells to DOX treatment also involves activation of Bax, cytochrome c release, and activation of caspase 3. A similar cooperative proapoptotic effect has been observed in glioblastoma and neuroblastoma cells [24], [25]. In neuroblastoma, the cooperative proapoptotic effect of DOX and PI103 resulted in upregulation of Noxa and Bim, which correlated with increased Bax/Bak conformational change, loss of mitochondrial membrane potential, cytochrome c release, caspase activation, and caspase-dependent apoptosis [25]. Furthermore, as in our study on sarcoma, sensitization to DOX-induced apoptosis in glioblastoma was mainly due to inhibition of PI3K, but not of mTOR [24]. These data suggest that the molecular mechanism resulting in PI103-mediated sensitization of sarcoma to DOX is similar to the chemosensitization of neuroblastoma and glioblastoma and involves the activation of several proteins of the mitochondrial apoptosis pathway.

Since the reason for the combined effect of PI103 and DOX on the mitochondrial apoptosis pathway activation is still unknown, we addressed several questions: First, we wanted to know whether PI103-mediated inhibition of PI3K/Akt activity may decrease the expression of MDR1 and MRP1, ultimately resulting in accumulation of DOX in tumor cells. This was indeed the case as shown by our experiments. However, the accumulation did not enhance DOX mediated proapoptotic effects.

Secondly, we investigated whether the sensitization of PI103 to DOX-mediated apoptosis involved mTOR inhibition. Our experiments performed with the pure mTOR inhibitor everolimus clearly argues against a role of mTOR in the sensitization process, because this drug increased neither DOX-induced numbers of Annexin V-positive cells nor caspase 3 activity. Instead, everolimus induced Akt activity in RD cells.

Finally, we investigated whether the drug combination resulted in any cooperative proapoptotic and antitumoral effect *in vivo*. For combination treatment we used GDC-0941, which is an orally available PI3K inhibitor with almost no mTOR modulating effects. Indeed, the *in vivo* experiments demonstrated that the combination of GDC-0941 plus DOX significantly increased the numbers of caspase 3 positive cells. This indicates that PI3K inhibition in combination with DOX increases proapoptotic events also *in vivo*. Nevertheless, the increase in apoptosis did not further impact tumor growth or tumor regrowth when compared to GDC-0941 alone.

The lack of a distinct cooperative suppression of tumor growth by DOX plus GDC-0941 *in vivo* despite the synergistic induction of apoptosis *in vitro* and the increase in caspase 3 cleavage *in vivo* in response to the combination treatment points to a more complex setting *in vivo*. A possible explanation for the lack of a cooperative antitumoral effect may be factors provided by the tumor microenvironment that transiently or permanently influence the drug response of tumor cells. In addition, tumor hypoxia may have an impact on the efficacy of the combination treatment that becomes relevant *in vivo*. Reasons such as poor drug penetration into tumor cells due to limited resorption, inefficient transport to the tumor via blood vessels or inefficient transportation from the vessels into the tumor tissue are less likely because both DOX and GDC-0941 showed antitumor activity when applied alone. Additional studies, also using other orally available inhibitors of the PI3K axis, are required to answer the question whether DOX plus GDC-0941 act in concert to suppress RMS growth *in vivo.*

## Supporting Information

Figure S1 DOX plus PI103 results in induction of early and late apoptosis. Annexin V positive cells in the living cell fractions of the cell lines RD (**A**), TP5014 (**B**) and HT1080 (**C**) were distinguished according to the positivity of both Annexin V and PI. As demonstrated, the treatment of DOX plus PI103 increased the numbers of both early (Annexin V^+^ PI^−^) and late (Annexin V^+^ PI^+^) apoptotic cells.
(TIF)

Figure S2 Apoptosis induced by DOX plus PI103 is caspase-dependent. RD cells were treated for 24 h with 1 µM DOX or 3 µM PI103 or 1 µM DOX plus 3 µM PI103 with or without 20 µM of the broad-range caspase inhibitor zVAD.fmk. Apoptosis was analyzed by FACS of Annexin V positive cells. The data shows that zVAD.fmk blocked apoptosis upon combined treatment with DOX and PI103, demonstrating caspase dependency. Statistical difference was analyzed by Student’s *t*-test.
(TIF)

Figure S3 Pretreatment with PI103 further strengthened the antiproliferative effects of DOX. The preincubation of the cells for 12 h with 3 µM PI103 (“PI103 [3 µM]_preinc._”) resulted in a significant increase of the antiproliferative effect of 0.5 µM DOX in RD (**A**), TP5014 (**B**) and HT1080 (**C**) cells. In this setting, the antiproliferative effect was superior to that caused by a 24-hours co-incubation with the drugs in all three cell lines (please compare results to the BrdU data shown in Figure 1B of the main manuscript). Comparisons were made with ANOVA/Tukey’s testing. *P<0.05 compared to cells treated with solvent; #*P*<0.05 compared to cells treated with either drug alone.
(TIF)

Figure S4 Activation of Bax after treatment TP5014 and HT1080 cells with PI103 and/or DOX. TP5014 (**A**) and HT1080 (**B**) cells were treated with 3 µM PI103 and 1 µM DOX for 24 h and Bax activity was analysed by Western Blot. Whereas a treatment with DOX marginally changed the conformational stage of Bax in HT1080 cells, the effect on Bax activation was enhanced in both cell lines when the drugs were combined.
(TIF)

Figure S5 GDC-0941 sensitizes RD cells to DOX-induced apoptosis. RD cells were treated for 48 h with 1 µM DOX or 10 µM GDC-0941 or 1 µM DOX plus 10 µM GDC-0941. Apoptosis was analyzed by FACS of Annexin V positive cells. The data shows that GDC-0941 sensitizes RD cells to DOX-induced apoptosis. Data represent mean+SEM of one experiment performed in triplicates. Comparisons were made with ANOVA/Tukey’s testing. *P<0.05 compared to cells treated with solvent; #*P*<0.05 compared to cells treated with either drug alone.
(TIF)

Table S1 Primary and secondary antibodies for Western Blot.
(DOC)

Table S2 Significance of tumor growth inhibition after treatment with either DOX (1.2 mg/kg), GDC-0941 (75 mg/kg or 25 mg/kg) or combination of the drugs. Sizes of 18 vehicle-treated, 19 DOX-treated, 9 GDC-0941-treated and 12 DOX plus GDC-0941-treated tumors were used to calculate the significance of changes in growth after a 7, 14 or 21 days treatment with 75 mg/kg GDC-0941 and/or 1.2 mg/kg DOX. For the study using 25 mg/kg GDC-0941 and/or 1.2 mg/kg DOX the sizes of 4 vehicle-treated, 6 DOX-treated, 6 GDC-0941-treated and 6 DOX plus GDC-0941-treated tumors were used to calculate the significance of changes in growth after a 7 and 14 days treatment with the drugs. *P* values were calculated by ANOVA/Tukey’s method and adjusted for tumor size differences at the onset of the treatment.
(DOC)

Table S3 Significance of tumor regrowth after treatment with either DOX (1.2 mg/kg), GDC-0941 (75 mg/kg) or combination of the drugs. Sizes of 6 vehicle-treated, 4 DOX-treated, 4 GDC-0941-treated and 4 DOX plus GDC-0941-treated tumors were used to calculate the significance of changes in tumor regrowth after a 21 days treatment with 75 mg/kg GDC-0941 and/or 1.2 mg/kg DOX. The observation period after treatment end was 20 days. *P* values were calculated by ANOVA/Tukey’s method and adjusted for tumor size differences at the onset of the treatment.
(DOC)
