# Supplementary figures and images for: PI3K Inhibition Enhances Doxorubicin-Induced Apoptosis in Sarcoma Cells
Source: PLoS One. 2012 Dec 31;7(12):e52898. doi: 10.1371/journal.pone.0052898 (PMC3534123; doi:10.1371/journal.pone.0052898)

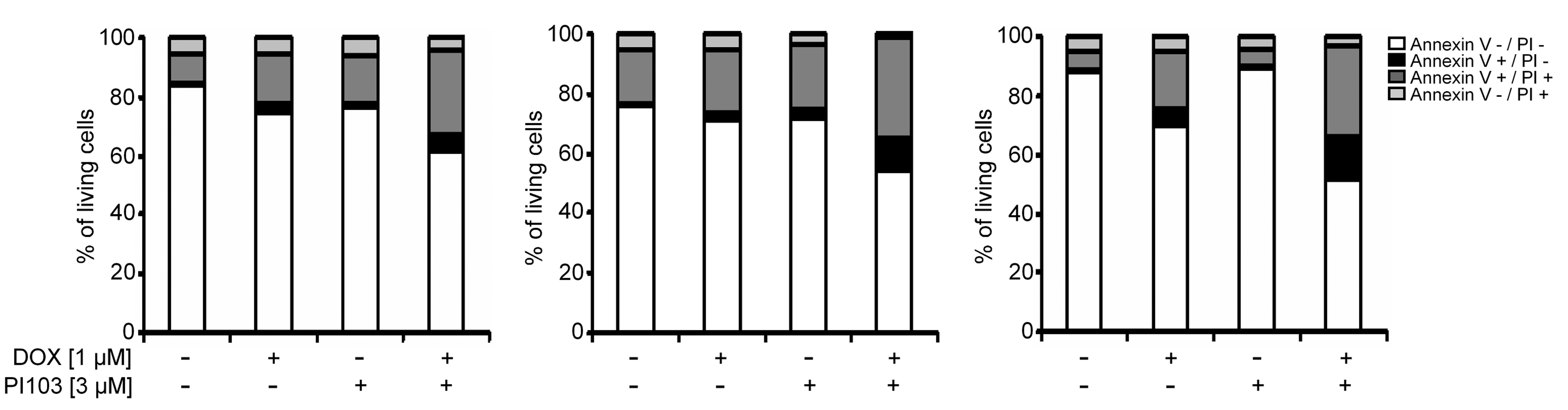

Supplement: Figure S1 — DOX plus PI103 results in induction of early and late apoptosis. Annexin V positive cells in the living cell fractions of the cell lines RD (A), TP5014 (B) and HT1080 (C) were distinguished according to the positivity of both Annexin V and PI. As demonstrated, the treatment of DOX plus PI103 increased the numbers of both early (Annexin V+ PI−) and late (Annexin V+ PI+) apoptotic cells. (TIF) [file pone.0052898.s001.tif]

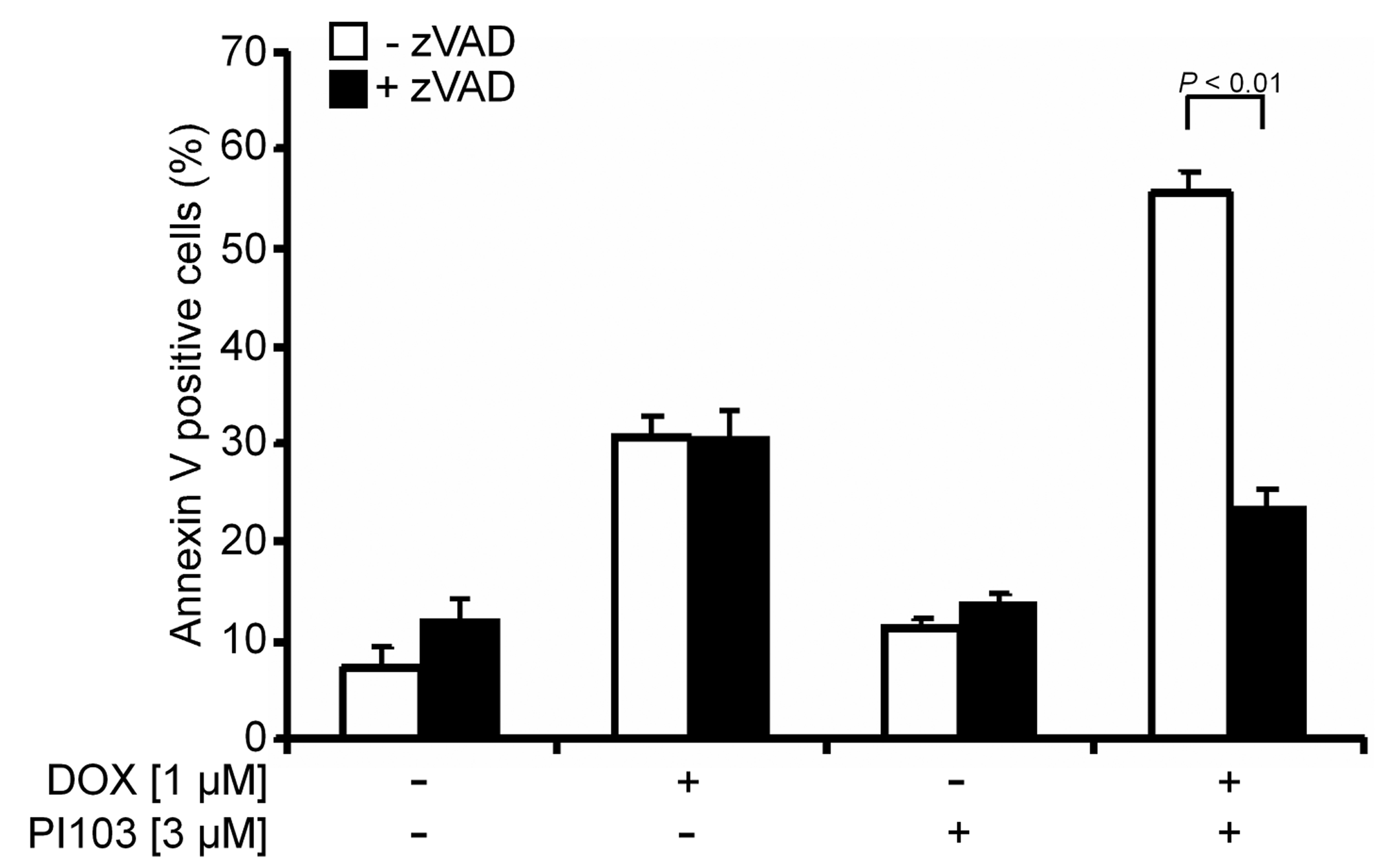

Supplement: Figure S2 — Apoptosis induced by DOX plus PI103 is caspase-dependent. RD cells were treated for 24 h with 1 µM DOX or 3 µM PI103 or 1 µM DOX plus 3 µM PI103 with or without 20 µM of the broad-range caspase inhibitor zVAD.fmk. Apoptosis was analyzed by FACS of Annexin V positive cells. The data shows that zVAD.fmk blocked apoptosis upon combined treatment with DOX and PI103, demonstrating caspase dependency. Statistical difference was analyzed by Student’s t-test. (TIF) [file pone.0052898.s002.tif]

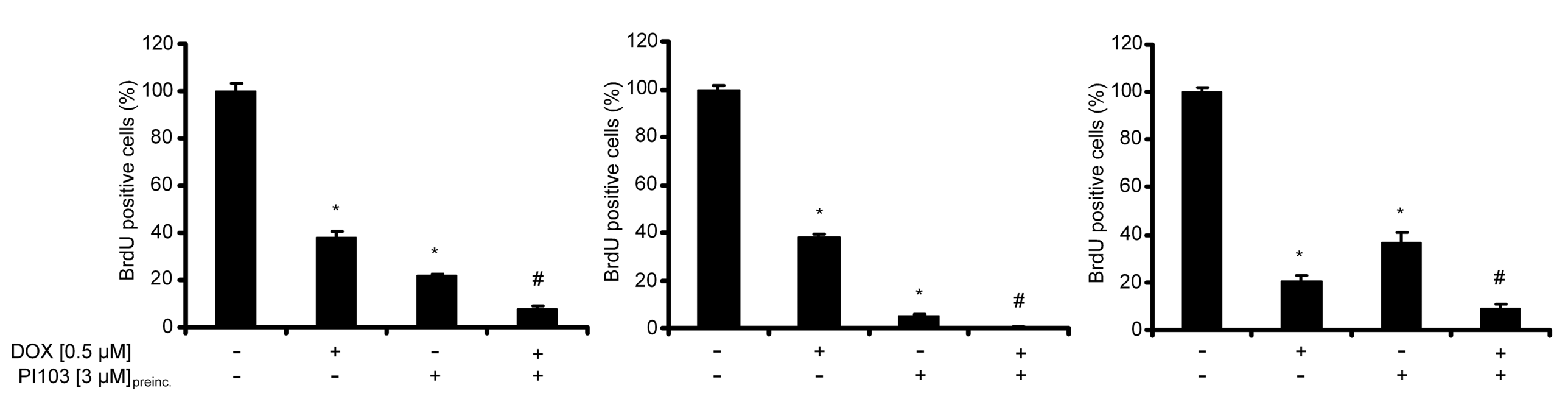

Supplement: Figure S3 — Pretreatment with PI103 further strengthened the antiproliferative effects of DOX. The preincubation of the cells for 12 h with 3 µM PI103 (“PI103 [3 µM]preinc.”) resulted in a significant increase of the antiproliferative effect of 0.5 µM DOX in RD (A), TP5014 (B) and HT1080 (C) cells. In this setting, the antiproliferative effect was superior to that caused by a 24-hours co-incubation with the drugs in all three cell lines (please compare results to the BrdU data shown in Figure 1B of the main manuscript). Comparisons were made with ANOVA/Tukey’s testing. *P<0.05 compared to cells treated with solvent; #P<0.05 compared to cells treated with either drug alone. (TIF) [file pone.0052898.s003.tif]

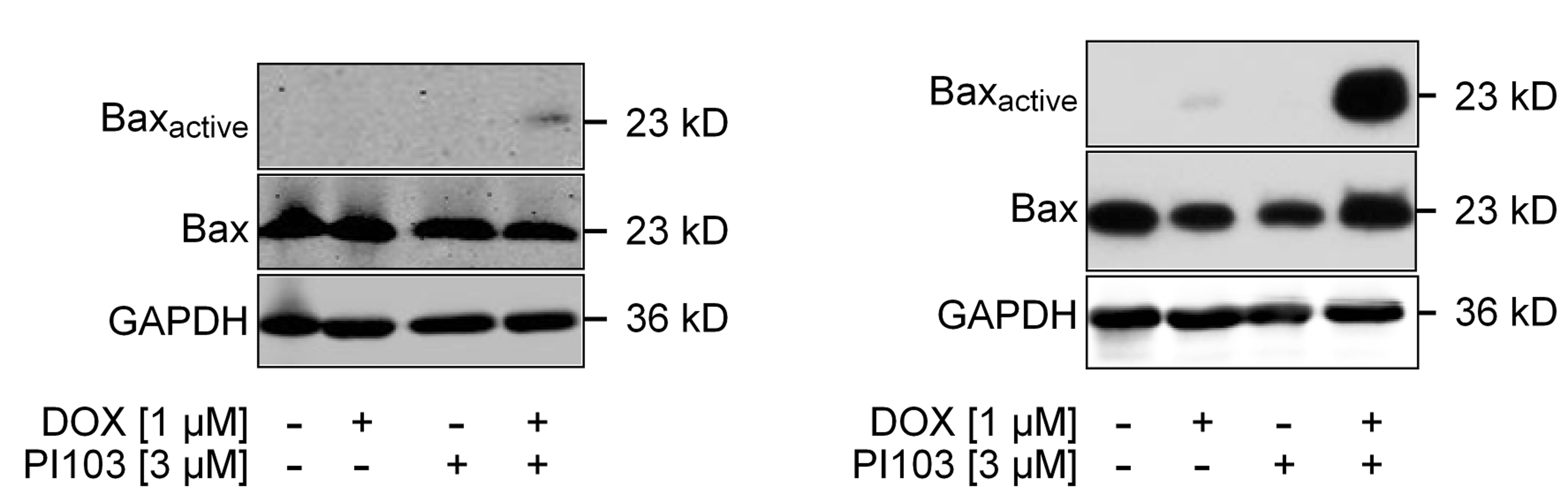

Supplement: Figure S4 — Activation of Bax after treatment TP5014 and HT1080 cells with PI103 and/or DOX. TP5014 (A) and HT1080 (B) cells were treated with 3 µM PI103 and 1 µM DOX for 24 h and Bax activity was analysed by Western Blot. Whereas a treatment with DOX marginally changed the conformational stage of Bax in HT1080 cells, the effect on Bax activation was enhanced in both cell lines when the drugs were combined. (TIF) [file pone.0052898.s004.tif]
