# Supplementary material for: PI3K Inhibition Enhances Doxorubicin-Induced Apoptosis in Sarcoma Cells
Source: PLoS One. 2012 Dec 31;7(12):e52898. doi: 10.1371/journal.pone.0052898 (PMC3534123; doi:10.1371/journal.pone.0052898)
Supplement: Table S1 — Primary and secondary antibodies for Western Blot. (DOC) [file pone.0052898.s006.doc]

**Table S1**

| **Primary antibodies for Western Blot** | **Dilution in PBS-T** |
| --- | --- |
| mAB mouse anti-Akt;610861; BD Biosciences Pharmingen | 1:1000 |
| mAB rabbit anti-pAkt (Ser473), 4058, Cell Signaling | 1:1000 |
| mAB mouse anti-Bax 6A7, B 8429, Sigma-Aldrich | 1:400 |
| pAB rabbit anti-BaxNT, 06-499, Upstate Biotechnology | 1:5000 |
| mAB mouse anti-β-Aktin, 4970, Cell Signaling | 1:10000 |
| pAB, rabbit anti-Caspase 3, 9662, Cell Signaling | 1:1000 |
| mAB mouse Anti-GAPDH, 5G4, HyTest Ltd | 1:5000 |
| mAB mouse anti-HSC70, sc-7298, Santa Cruz | 1:10000 |
| mAB mouse anti-MRP1, ab32574, Abcam | 1:500 |
| pAB rabbit anti-pS6, 2215, Cell Signaling | 1:1000 |
| mAB mouse anti-S6, 2317, Cell Signaling | 1:1000 |
| mAB mouse anti-α-Tubulin, DLN-09992, Dianova | 1:10000 |
| **Secondary antibodies for western Blot** | **Dilution in PBS-T** |
| pAB sheep anti-mouse/HRP, NA931, GE Halthcare | 1:5000 |
| pAB goat anti-rabbit/HRP, A0545, Sigma-Aldrich | 1:5000 |
