# Supplementary material for: PI3K Inhibition Enhances Doxorubicin-Induced Apoptosis in Sarcoma Cells
Source: PLoS One. 2012 Dec 31;7(12):e52898. doi: 10.1371/journal.pone.0052898 (PMC3534123; doi:10.1371/journal.pone.0052898)
Supplement: Table S2 — Significance of tumor growth inhibition after treatment with either DOX (1.2 mg/kg), GDC-0941 (75 mg/kg or 25 mg/kg) or combination of the drugs. Sizes of 18 vehicle-treated, 19 DOX-treated, 9 GDC-0941-treated and 12 DOX plus GDC-0941-treated tumors were used to calculate the significance of changes in growth after a 7, 14 or 21 days treatment with 75 mg/kg GDC-0941 and/or 1.2 mg/kg DOX. For the study using 25 mg/kg GDC-0941 and/or 1.2 mg/kg DOX the sizes of 4 vehicle-treated, 6 DOX-treated, 6 GDC-0941-treated and 6 DOX plus GDC-0941-treated tumors were used to calculate the significance of changes in growth after a 7 and 14 days treatment with the drugs. P values were calculated by ANOVA/Tukey’s method and adjusted for tumor size differences at the onset of the treatment. (DOC) [file pone.0052898.s007.doc]

**Table S2**

|  |  | **75 mg/kg GDC-0941 plus 1.2 mg/kg DOX** | | | **25 mg/kg GDC-0941 plus 1.2 mg/kg DOX** | | |
| --- | --- | --- | --- | --- | --- | --- | --- |
| Days of treatment |  | GDC-0941 | vehicle | DOX | GDC-0941 | vehicle | DOX |
| 7 | DOX/GDC-0941 | 0.9866 | 0.4221 | 0.1603 | 0.8018 | 0.0003 | 0.9997 |
|  | GDC-0941 | **–** | 0.2924 | 0.1045 |  | 0.2261 | 0.8324 |
|  | vehicle | **–** | **–** | 0.9171 |  |  | 0.6770 |
| 14 | DOX/GDC-0941 | 0.9422 | <0.0001 | 0.0011 | 0.1898 | 0.0619 | 0.9849 |
|  | GDC-0941 | **–** | 0.0002 | 0.0228 |  | 0.0205 | 0.3076 |
|  | vehicle | **–** | **–** | 0.3783 |  |  | 0.5737 |
| 21 | DOX/GDC-0941 | 0.4799 | <0.0001 | <0.0001 | **–** | **–** | **–** |
|  | GDC-0941 | **–** | <0.0001 | 0.0053 | **–** | **–** | **–** |
|  | vehicle | **–** | **–** | 0.0011 | **–** | **–** | **–** |
