# Supplementary material for: PI3K Inhibition Enhances Doxorubicin-Induced Apoptosis in Sarcoma Cells
Source: PLoS One. 2012 Dec 31;7(12):e52898. doi: 10.1371/journal.pone.0052898 (PMC3534123; doi:10.1371/journal.pone.0052898)
Supplement: Table S3 — Significance of tumor regrowth after treatment with either DOX (1.2 mg/kg), GDC-0941 (75 mg/kg) or combination of the drugs. Sizes of 6 vehicle-treated, 4 DOX-treated, 4 GDC-0941-treated and 4 DOX plus GDC-0941-treated tumors were used to calculate the significance of changes in tumor regrowth after a 21 days treatment with 75 mg/kg GDC-0941 and/or 1.2 mg/kg DOX. The observation period after treatment end was 20 days. P values were calculated by ANOVA/Tukey’s method and adjusted for tumor size differences at the onset of the treatment. (DOC) [file pone.0052898.s008.doc]

**Table S3**

| Days after end of treatment |  | GDC-0941 | vehicle | DOX |
| --- | --- | --- | --- | --- |
| 10 | DOX/GDC-0941 | 0.9274 | 0.9687 | 0.8266 |
|  | GDC-0941 | **–** | 0.9961 | 0.4796 |
|  | vehicle | **–** | **–** | 0.5231 |
| 20 | DOX/GDC-0941 | 0.9255 | 0.9041 | 0.418 |
|  | GDC-0941 | **–** | 1.0000 | 0.1653 |
|  | vehicle | **–** | **–** | 0.1136 |
